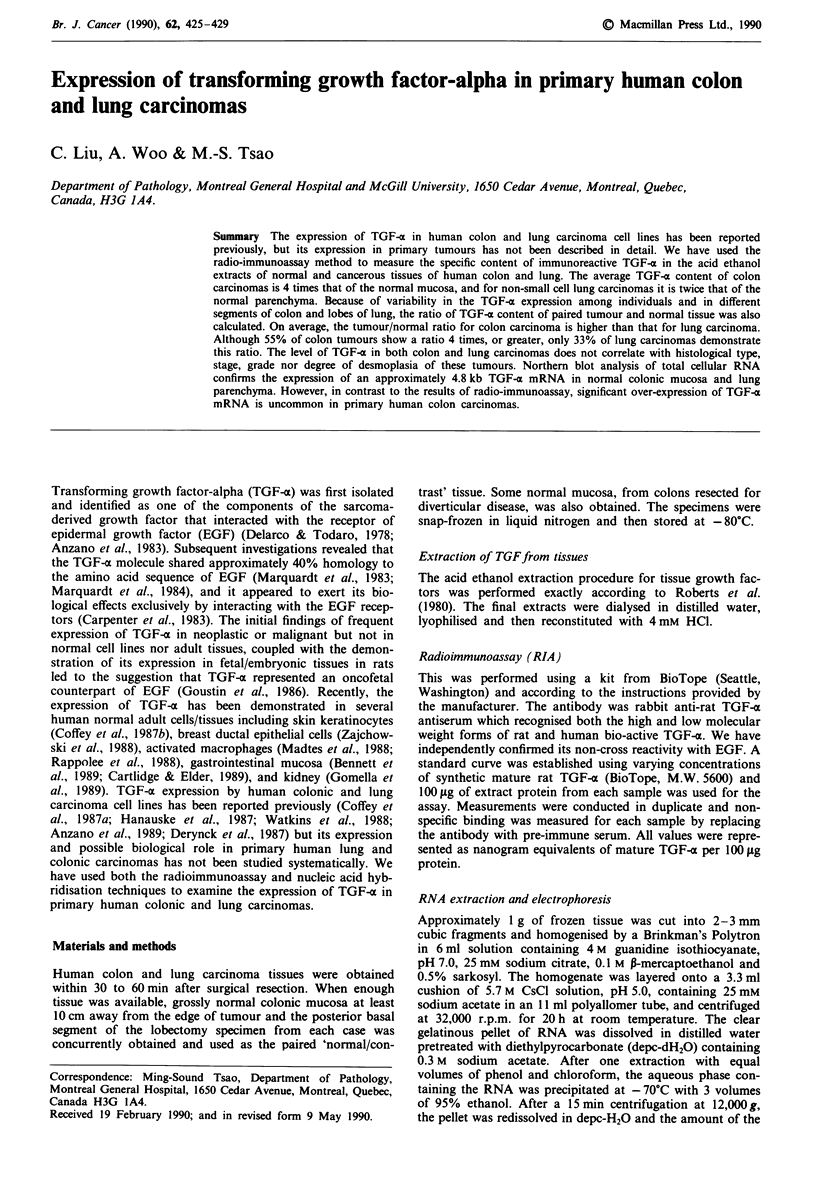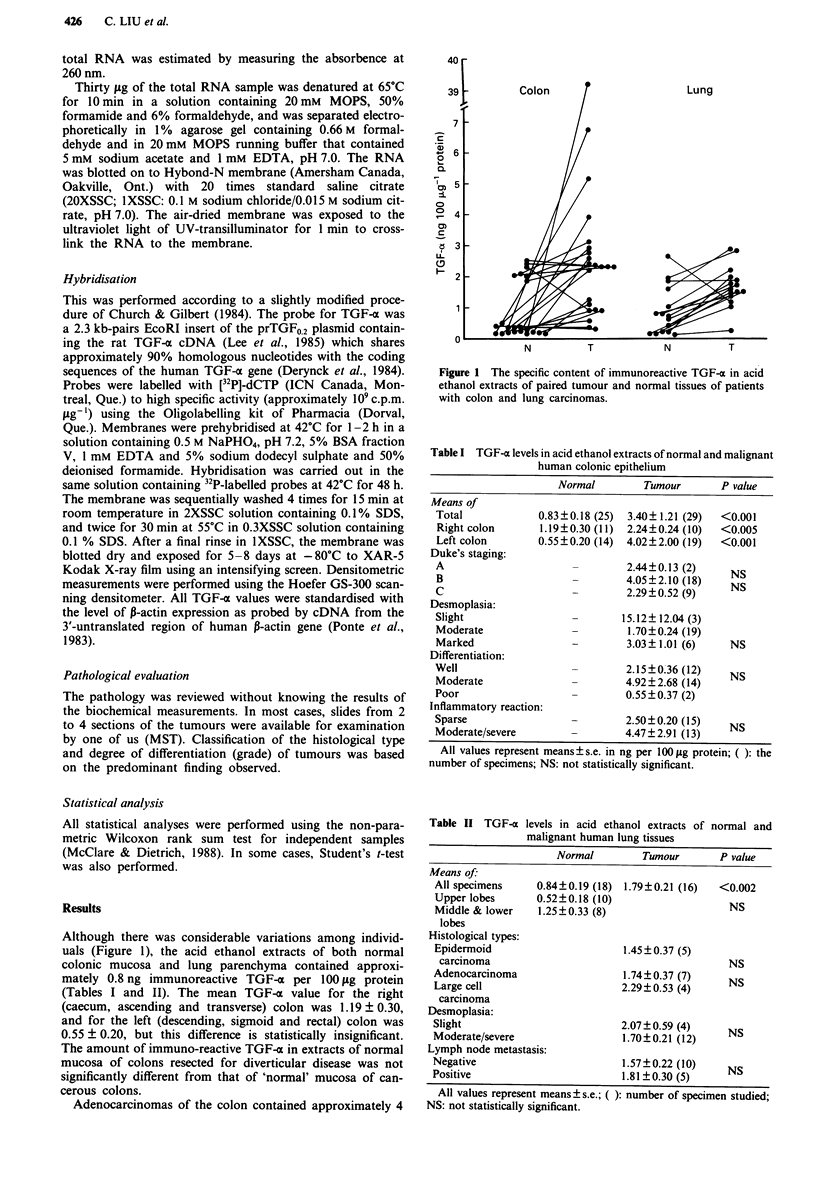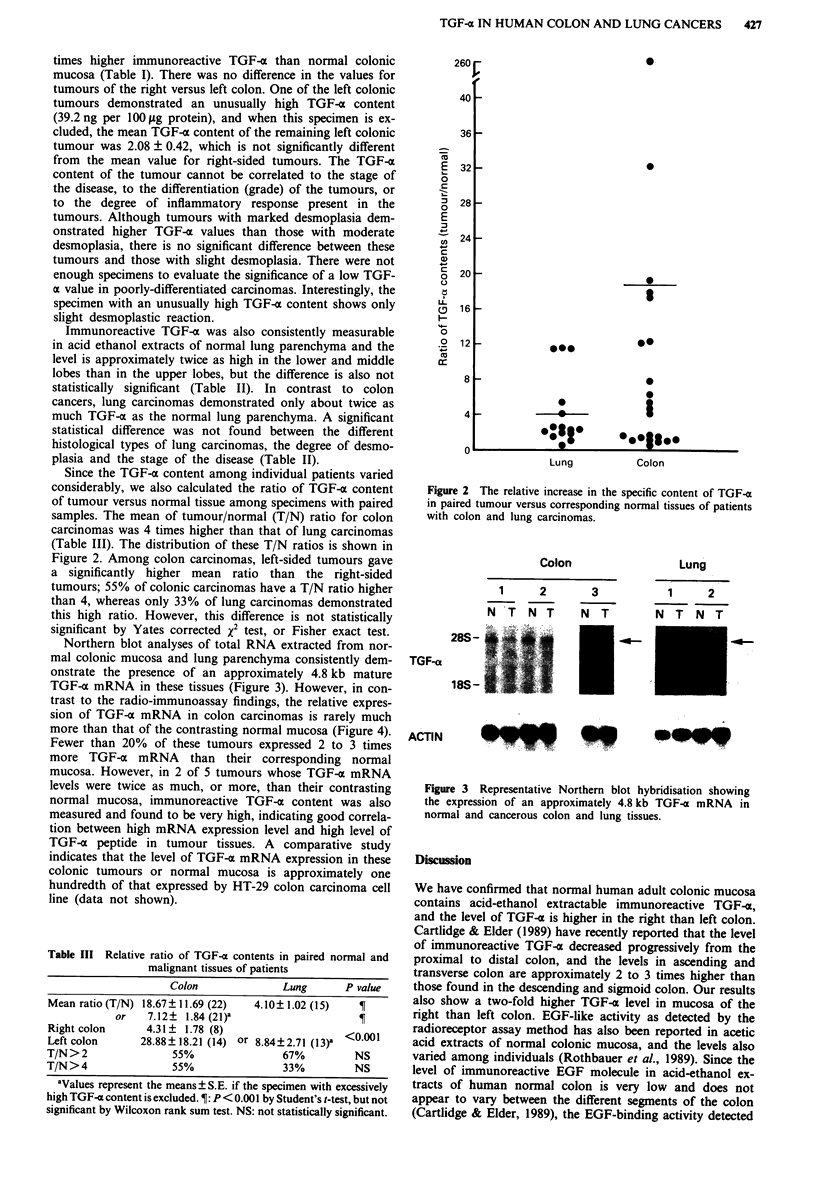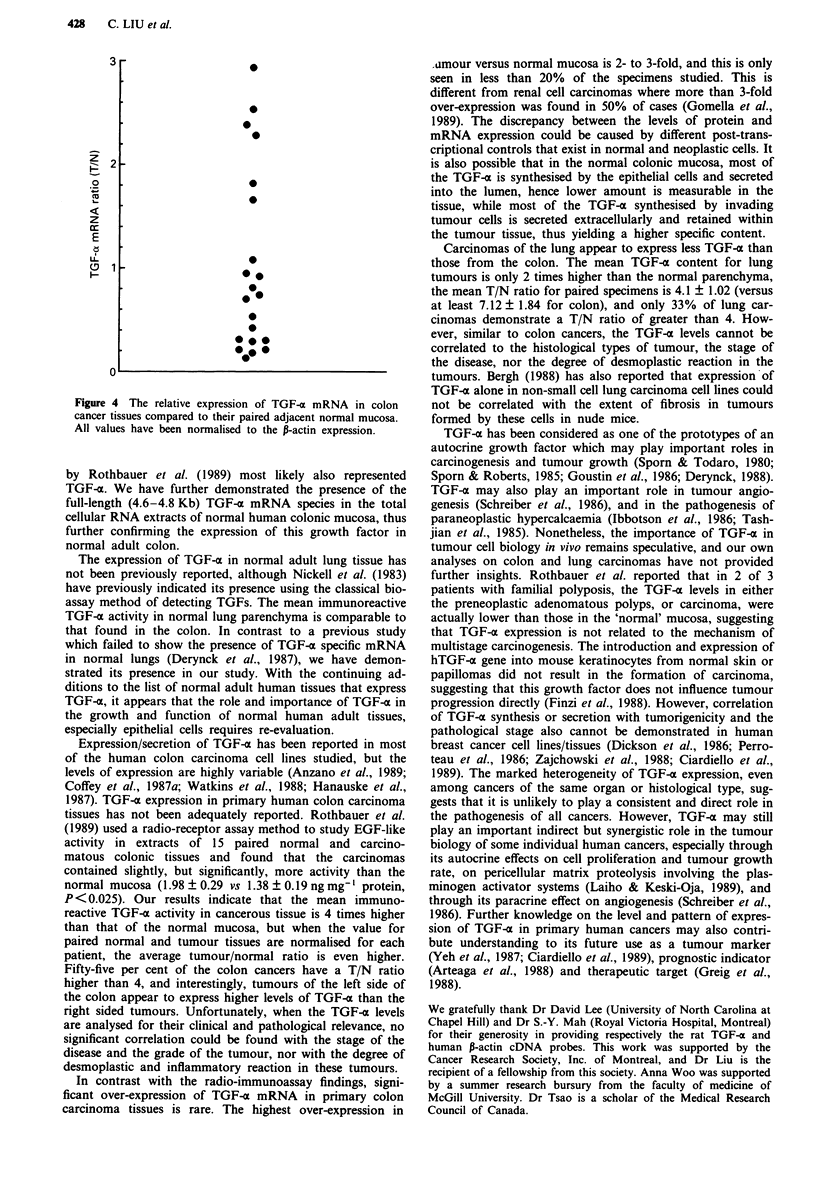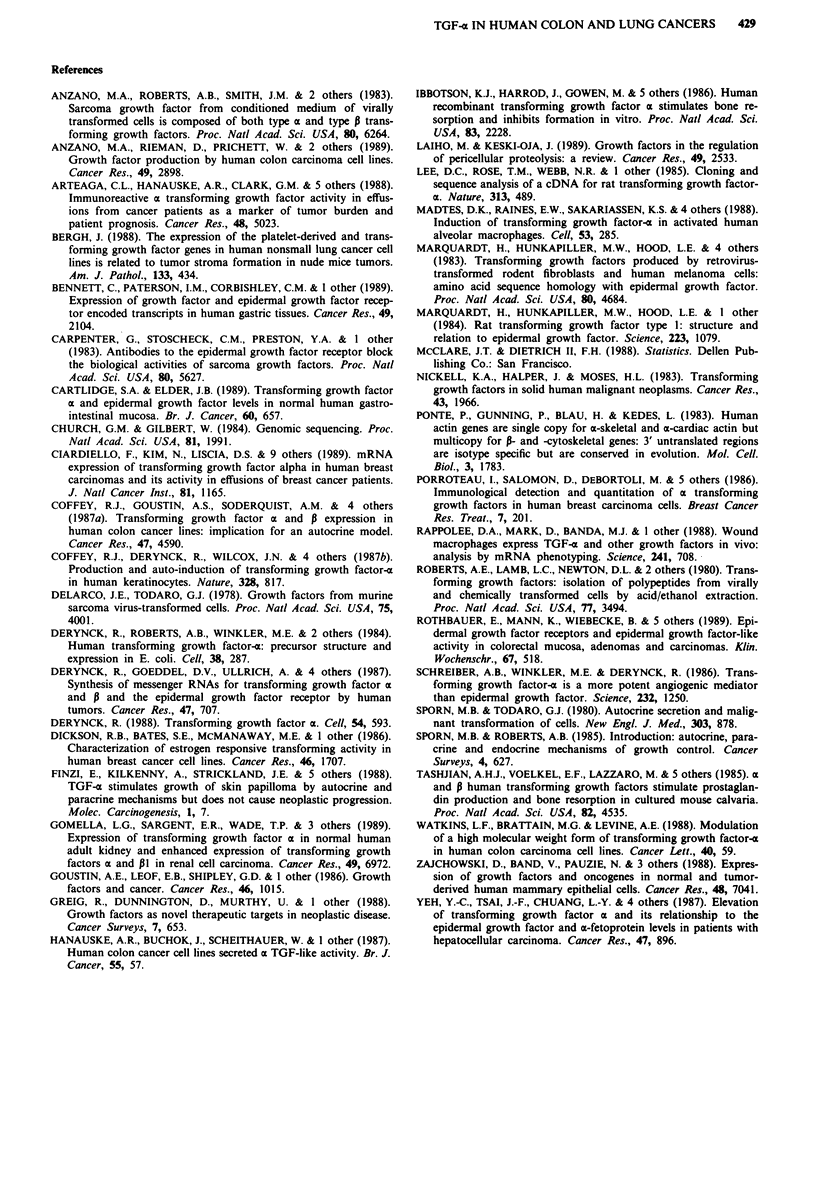# Expression of transforming growth factor-alpha in primary human colon and lung carcinomas.

**DOI:** 10.1038/bjc.1990.311

**Published:** 1990-09

**Authors:** C. Liu, A. Woo, M. S. Tsao

**Affiliations:** Department of Pathology, Montreal General Hospital, Quebec, Canada.

## Abstract

**Images:**


Br. J. Cancer (1990), 62, 425–429 © Macmillan Press Ltd., 1990

### Expression of transforming growth factor-alpha in primary human colon and lung carcinomas

C. Liu, A. Woo & M.-S. Tsao

*Department of Pathology, Montreal General Hospital and McGill University, 1650 Cedar Avenue, Montreal, Quebec, Canada, H3G 1A4.*

**Summary** The expression of TGF-α in human colon and lung carcinoma cell lines has been reported previously, but its expression in primary tumours has not been described in detail. We have used the radio-immunoassay method to measure the specific content of immunoreactive TGF-α in the acid ethanol extracts of normal and cancerous tissues of human colon and lung. The average TGF-α content of colon carcinomas is 4 times that of the normal mucosa, and for non-small cell lung carcinomas it is twice that of the normal parenchyma. Because of variability in the TGF-α expression among individuals and in different segments of colon and lobes of lung, the ratio of TGF-α content of paired tumour and normal tissue was also calculated. On average, the tumour/normal ratio for colon carcinoma is higher than that for lung carcinoma. Although 55% of colon tumours show a ratio 4 times, or greater, only 33% of lung carcinomas demonstrate this ratio. The level of TGF-α in both colon and lung carcinomas does not correlate with histological type, stage, grade nor degree of desmoplasia of these tumours. Northern blot analysis of total cellular RNA confirms the expression of an approximately 4.8 kb TGF-α mRNA in normal colonic mucosa and lung parenchyma. However, in contrast to the results of radio-immunoassay, significant over-expression of TGF-α mRNA is uncommon in primary human colon carcinomas.

Transforming growth factor-alpha (TGF-α) was first isolated and identified as one of the components of the sarcoma-derived growth factor that interacted with the receptor of epidermal growth factor (EGF) (Delarco & Todaro, 1978; Anzano *et al.*, 1983). Subsequent investigations revealed that the TGF-α molecule shared approximately 40% homology to the amino acid sequence of EGF (Marquardt *et al.*, 1983; Marquardt *et al.*, 1984), and it appeared to exert its biological effects exclusively by interacting with the EGF receptors (Carpenter *et al.*, 1983). The initial findings of frequent expression of TGF-α in neoplastic or malignant but not in normal cell lines nor adult tissues, coupled with the demonstration of its expression in fetal/embryonic tissues in rats led to the suggestion that TGF-α represented an oncofetal counterpart of EGF (Goustin *et al.*, 1986). Recently, the expression of TGF-α has been demonstrated in several human normal adult cells/tissues including skin keratinocytes (Coffey *et al.*, 1987*b*), breast ductal epithelial cells (Zajchowski *et al.*, 1988), activated macrophages (Madtes *et al.*, 1988; Rappolee *et al.*, 1988), gastrointestinal mucosa (Bennett *et al.*, 1989; Cartlidge & Elder, 1989), and kidney (Gomella *et al.*, 1989). TGF-α expression by human colonic and lung carcinoma cell lines has been reported previously (Coffey *et al.*, 1987*a*; Hanauske *et al.*, 1987; Watkins *et al.*, 1988; Anzano *et al.*, 1989; Derynck *et al.*, 1987) but its expression and possible biological role in primary human lung and colonic carcinomas has not been studied systematically. We have used both the radioimmunoassay and nucleic acid hybridisation techniques to examine the expression of TGF-α in primary human colonic and lung carcinomas.

### Materials and methods

Human colon and lung carcinoma tissues were obtained within 30 to 60 min after surgical resection. When enough tissue was available, grossly normal colonic mucosa at least 10 cm away from the edge of tumour and the posterior basal segment of the lobectomy specimen from each case was concurrently obtained and used as the paired 'normal/contrast' tissue. Some normal mucosa, from colons resected for diverticular disease, was also obtained. The specimens were snap-frozen in liquid nitrogen and then stored at −80°C.

Correspondence: Ming-Sound Tsao, Department of Pathology, Montreal General Hospital, 1650 Cedar Avenue, Montreal, Quebec, Canada H3G 1A4.
Received 19 February 1990; and in revised form 9 May 1990.

### *Extraction of TGF from tissues*

The acid ethanol extraction procedure for tissue growth factors was performed exactly according to Roberts *et al.* (1980). The final extracts were dialysed in distilled water, lyophilised and then reconstituted with 4 mM HCl.

### *Radioimmunoassay (RIA)*

This was performed using a kit from BioTope (Seattle, Washington) and according to the instructions provided by the manufacturer. The antibody was rabbit anti-rat TGF-α antiserum which recognised both the high and low molecular weight forms of rat and human bio-active TGF-α. We have independently confirmed its non-cross reactivity with EGF. A standard curve was established using varying concentrations of synthetic mature rat TGF-α (BioTope, M.W. 5600) and 100 μg of extract protein from each sample was used for the assay. Measurements were conducted in duplicate and non-specific binding was measured for each sample by replacing the antibody with pre-immune serum. All values were represented as nanogram equivalents of mature TGF-α per 100 μg protein.

### *RNA extraction and electrophoresis*

Approximately 1 g of frozen tissue was cut into 2–3 mm cubic fragments and homogenised by a Brinkman's Polytron in 6 ml solution containing 4 M guanidine isothiocyanate, pH 7.0, 25 mM sodium citrate, 0.1 M β-mercaptoethanol and 0.5% sarkosyl. The homogenate was layered onto a 3.3 ml cushion of 5.7 M CsCl solution, pH 5.0, containing 25 mM sodium acetate in an 11 ml polyallomer tube, and centrifuged at 32,000 r.p.m. for 20 h at room temperature. The clear gelatinous pellet of RNA was dissolved in distilled water pretreated with diethylpyrocarbonate (depc-d$H_2O$) containing 0.3 M sodium acetate. After one extraction with equal volumes of phenol and chloroform, the aqueous phase containing the RNA was precipitated at −70°C with 3 volumes of 95% ethanol. After a 15 min centrifugation at 12,000 *g*, the pellet was redissolved in depc-$H_2O$ and the amount of the total RNA was estimated by measuring the absorbence at 260 nm.

Thirty µg of the total RNA sample was denatured at 65°C for 10 min in a solution containing 20 mM MOPS, 50% formamide and 6% formaldehyde, and was separated electrophoretically in 1% agarose gel containing 0.66 M formaldehyde and in 20 mM MOPS running buffer that contained 5 mM sodium acetate and 1 mM EDTA, pH 7.0. The RNA was blotted on to Hybond-N membrane (Amersham Canada, Oakville, Ont.) with 20 times standard saline citrate (20XSSC; 1XSSC: 0.1 M sodium chloride/0.015 M sodium citrate, pH 7.0). The air-dried membrane was exposed to the ultraviolet light of UV-transilluminator for 1 min to cross-link the RNA to the membrane.

### *Hybridisation*

This was performed according to a slightly modified procedure of Church & Gilbert (1984). The probe for TGF-α was a 2.3 kb-pairs EcoRI insert of the $prTGF_{0.2}$ plasmid containing the rat TGF-α cDNA (Lee *et al.*, 1985) which shares approximately 90% homologous nucleotides with the coding sequences of the human TGF-α gene (Derynck *et al.*, 1984). Probes were labelled with [$^{32}$P]-dCTP (ICN Canada, Montreal, Que.) to high specific activity (approximately $10^9$ c.p.m. $\mu g^{-1}$) using the Oligolabelling kit of Pharmacia (Dorval, Que.). Membranes were prehybridised at 42°C for 1–2 h in a solution containing 0.5 M $NaPHO_4$, pH 7.2, 5% BSA fraction V, 1 mM EDTA and 5% sodium dodecyl sulphate and 50% deionised formamide. Hybridisation was carried out in the same solution containing $^{32}$P-labelled probes at 42°C for 48 h. The membrane was sequentially washed 4 times for 15 min at room temperature in 2XSSC solution containing 0.1% SDS, and twice for 30 min at 55°C in 0.3XSSC solution containing 0.1 % SDS. After a final rinse in 1XSSC, the membrane was blotted dry and exposed for 5–8 days at −80°C to XAR-5 Kodak X-ray film using an intensifying screen. Densitometric measurements were performed using the Hoefer GS-300 scanning densitometer. All TGF-α values were standardised with the level of β-actin expression as probed by cDNA from the 3'-untranslated region of human β-actin gene (Ponte *et al.*, 1983).

### *Pathological evaluation*

The pathology was reviewed without knowing the results of the biochemical measurements. In most cases, slides from 2 to 4 sections of the tumours were available for examination by one of us (MST). Classification of the histological type and degree of differentiation (grade) of tumours was based on the predominant finding observed.

### *Statistical analysis*

All statistical analyses were performed using the non-parametric Wilcoxon rank sum test for independent samples (McClare & Dietrich, 1988). In some cases, Student's *t*-test was also performed.

### Results

Although there was considerable variations among individuals (Figure 1), the acid ethanol extracts of both normal colonic mucosa and lung parenchyma contained approximately 0.8 ng immunoreactive TGF-α per 100 µg protein (Tables I and II). The mean TGF-α value for the right (caecum, ascending and transverse) colon was 1.19 ± 0.30, and for the left (descending, sigmoid and rectal) colon was 0.55 ± 0.20, but this difference is statistically insignificant. The amount of immuno-reactive TGF-α in extracts of normal mucosa of colons resected for diverticular disease was not significantly different from that of 'normal' mucosa of cancerous colons.

Adenocarcinomas of the colon contained approximately 4

**Figure 1** The specific content of immunoreactive TGF-α in acid ethanol extracts of paired tumour and normal tissues of patients with colon and lung carcinomas.

**Table I** TGF-α levels in acid ethanol extracts of normal and malignant human colonic epithelium

| | *Normal* | *Tumour* | *P value* |
|---|---|---|---|
| *Means of* | | | |
| Total | 0.83±0.18 (25) | 3.40±1.21 (29) | <0.001 |
| Right colon | 1.19±0.30 (11) | 2.24±0.24 (10) | <0.005 |
| Left colon | 0.55±0.20 (14) | 4.02±2.00 (19) | <0.001 |
| Duke's staging: | | | |
| A | – | 2.44±0.13 (2) | |
| B | – | 4.05±2.10 (18) | NS |
| C | – | 2.29±0.52 (9) | NS |
| Desmoplasia: | | | |
| Slight | – | 15.12±12.04 (3) | |
| Moderate | – | 1.70±0.24 (19) | |
| Marked | – | 3.03±1.01 (6) | NS |
| Differentiation: | | | |
| Well | – | 2.15±0.36 (12) | |
| Moderate | – | 4.92±2.68 (14) | NS |
| Poor | – | 0.55±0.37 (2) | |
| Inflammatory reaction: | | | |
| Sparse | – | 2.50±0.20 (15) | NS |
| Moderate/severe | – | 4.47±2.91 (13) | |

All values represent means±s.e. in ng per 100 µg protein; ( ): the number of specimens; NS: not statistically significant.

**Table II** TGF-α levels in acid ethanol extracts of normal and malignant human lung tissues

| | *Normal* | *Tumour* | *P value* |
|---|---|---|---|
| *Means of:* | | | |
| All specimens | 0.84±0.19 (18) | 1.79±0.21 (16) | <0.002 |
| Upper lobes | 0.52±0.18 (10) | | |
| Middle & lower lobes | 1.25±0.33 (8) | | NS |
| Histological types: | | | |
| Epidermoid carcinoma | | 1.45±0.37 (5) | NS |
| Adenocarcinoma | | 1.74±0.37 (7) | |
| Large cell carcinoma | | 2.29±0.53 (4) | NS |
| Desmoplasia: | | | |
| Slight | | 2.07±0.59 (4) | |
| Moderate/severe | | 1.70±0.21 (12) | NS |
| Lymph node metastasis: | | | |
| Negative | | 1.57±0.22 (10) | |
| Positive | | 1.81±0.30 (5) | NS |

All values represent means±s.e.; ( ): number of specimen studied; NS: not statistically significant.

times higher immunoreactive TGF-α than normal colonic mucosa (Table I). There was no difference in the values for tumours of the right versus left colon. One of the left colonic tumours demonstrated an unusually high TGF-α content (39.2 ng per 100 μg protein), and when this specimen is excluded, the mean TGF-α content of the remaining left colonic tumour was 2.08 ± 0.42, which is not significantly different from the mean value for right-sided tumours. The TGF-α content of the tumour cannot be correlated to the stage of the disease, to the differentiation (grade) of the tumours, or to the degree of inflammatory response present in the tumours. Although tumours with marked desmoplasia demonstrated higher TGF-α values than those with moderate desmoplasia, there is no significant difference between these tumours and those with slight desmoplasia. There were not enough specimens to evaluate the significance of a low TGF-α value in poorly-differentiated carcinomas. Interestingly, the specimen with an unusually high TGF-α content shows only slight desmoplastic reaction.

Immunoreactive TGF-α was also consistently measurable in acid ethanol extracts of normal lung parenchyma and the level is approximately twice as high in the lower and middle lobes than in the upper lobes, but the difference is also not statistically significant (Table II). In contrast to colon cancers, lung carcinomas demonstrated only about twice as much TGF-α as the normal lung parenchyma. A significant statistical difference was not found between the different histological types of lung carcinomas, the degree of desmoplasia and the stage of the disease (Table II).

Since the TGF-α content among individual patients varied considerably, we also calculated the ratio of TGF-α content of tumour versus normal tissue among specimens with paired samples. The mean of tumour/normal (T/N) ratio for colon carcinomas was 4 times higher than that of lung carcinomas (Table III). The distribution of these T/N ratios is shown in Figure 2. Among colon carcinomas, left-sided tumours gave a significantly higher mean ratio than the right-sided tumours; 55% of colonic carcinomas have a T/N ratio higher than 4, whereas only 33% of lung carcinomas demonstrated this high ratio. However, this difference is not statistically significant by Yates corrected $\chi^2$ test, or Fisher exact test.

Northern blot analyses of total RNA extracted from normal colonic mucosa and lung parenchyma consistently demonstrate the presence of an approximately 4.8 kb mature TGF-α mRNA in these tissues (Figure 3). However, in contrast to the radio-immunoassay findings, the relative expression of TGF-α mRNA in colon carcinomas is rarely much more than that of the contrasting normal mucosa (Figure 4). Fewer than 20% of these tumours expressed 2 to 3 times more TGF-α mRNA than their corresponding normal mucosa. However, in 2 of 5 tumours whose TGF-α mRNA levels were twice as much, or more, than their contrasting normal mucosa, immunoreactive TGF-α content was also measured and found to be very high, indicating good correlation between high mRNA expression level and high level of TGF-α peptide in tumour tissues. A comparative study indicates that the level of TGF-α mRNA expression in these colonic tumours or normal mucosa is approximately one hundredth of that expressed by HT-29 colon carcinoma cell line (data not shown).

**Table III** Relative ratio of TGF-α contents in paired normal and malignant tissues of patients

| | Colon | Lung | P value |
|---|---|---|---|
| Mean ratio (T/N) | 18.67±11.69 (22) | 4.10±1.02 (15) | ¶ |
| or | 7.12± 1.84 (21)[a] | | ¶ |
| Right colon | 4.31± 1.78 (8) | or 8.84±2.71 (13)[a] | <0.001 |
| Left colon | 28.88±18.21 (14) | | |
| T/N>2 | 55% | 67% | NS |
| T/N>4 | 55% | 33% | NS |

[a]Values represent the means±S.E. if the specimen with excessively high TGF-α content is excluded. ¶: $P < 0.001$ by Student's *t*-test, but not significant by Wilcoxon rank sum test. NS: not statistically significant.

**Figure 2** The relative increase in the specific content of TGF-α in paired tumour versus corresponding normal tissues of patients with colon and lung carcinomas.

**Figure 3** Representative Northern blot hybridisation showing the expression of an approximately 4.8 kb TGF-α mRNA in normal and cancerous colon and lung tissues.

### Discussion

We have confirmed that normal human adult colonic mucosa contains acid-ethanol extractable immunoreactive TGF-α, and the level of TGF-α is higher in the right than left colon. Cartlidge & Elder (1989) have recently reported that the level of immunoreactive TGF-α decreased progressively from the proximal to distal colon, and the levels in ascending and transverse colon are approximately 2 to 3 times higher than those found in the descending and sigmoid colon. Our results also show a two-fold higher TGF-α level in mucosa of the right than left colon. EGF-like activity as detected by the radioreceptor assay method has also been reported in acetic acid extracts of normal colonic mucosa, and the levels also varied among individuals (Rothbauer *et al.*, 1989). Since the level of immunoreactive EGF molecule in acid-ethanol extracts of human normal colon is very low and does not appear to vary between the different segments of the colon (Cartlidge & Elder, 1989), the EGF-binding activity detected

**Figure 4** The relative expression of TGF-α mRNA in colon cancer tissues compared to their paired adjacent normal mucosa. All values have been normalised to the β-actin expression.

by Rothbauer *et al.* (1989) most likely also represented TGF-α. We have further demonstrated the presence of the full-length (4.6–4.8 Kb) TGF-α mRNA species in the total cellular RNA extracts of normal human colonic mucosa, thus further confirming the expression of this growth factor in normal adult colon.

The expression of TGF-α in normal adult lung tissue has not been previously reported, although Nickell *et al.* (1983) have previously indicated its presence using the classical bioassay method of detecting TGFs. The mean immunoreactive TGF-α activity in normal lung parenchyma is comparable to that found in the colon. In contrast to a previous study which failed to show the presence of TGF-α specific mRNA in normal lungs (Derynck *et al.*, 1987), we have demonstrated its presence in our study. With the continuing additions to the list of normal adult human tissues that express TGF-α, it appears that the role and importance of TGF-α in the growth and function of normal human adult tissues, especially epithelial cells requires re-evaluation.

Expression/secretion of TGF-α has been reported in most of the human colon carcinoma cell lines studied, but the levels of expression are highly variable (Anzano *et al.*, 1989; Coffey *et al.*, 1987*a*; Watkins *et al.*, 1988; Hanauske *et al.*, 1987). TGF-α expression in primary human colon carcinoma tissues has not been adequately reported. Rothbauer *et al.* (1989) used a radio-receptor assay method to study EGF-like activity in extracts of 15 paired normal and carcinomatous colonic tissues and found that the carcinomas contained slightly, but significantly, more activity than the normal mucosa ($1.98 \pm 0.29$ *vs* $1.38 \pm 0.19$ ng mg$^{-1}$ protein, $P < 0.025$). Our results indicate that the mean immunoreactive TGF-α activity in cancerous tissue is 4 times higher than that of the normal mucosa, but when the value for paired normal and tumour tissues are normalised for each patient, the average tumour/normal ratio is even higher. Fifty-five per cent of the colon cancers have a T/N ratio higher than 4, and interestingly, tumours of the left side of the colon appear to express higher levels of TGF-α than the right sided tumours. Unfortunately, when the TGF-α levels are analysed for their clinical and pathological relevance, no significant correlation could be found with the stage of the disease and the grade of the tumour, nor with the degree of desmoplastic and inflammatory reaction in these tumours.

In contrast with the radio-immunoassay findings, significant over-expression of TGF-α mRNA in primary colon carcinoma tissues is rare. The highest over-expression in tumour versus normal mucosa is 2- to 3-fold, and this is only seen in less than 20% of the specimens studied. This is different from renal cell carcinomas where more than 3-fold over-expression was found in 50% of cases (Gomella *et al.*, 1989). The discrepancy between the levels of protein and mRNA expression could be caused by different post-transcriptional controls that exist in normal and neoplastic cells. It is also possible that in the normal colonic mucosa, most of the TGF-α is synthesised by the epithelial cells and secreted into the lumen, hence lower amount is measurable in the tissue, while most of the TGF-α synthesised by invading tumour cells is secreted extracellularly and retained within the tumour tissue, thus yielding a higher specific content.

Carcinomas of the lung appear to express less TGF-α than those from the colon. The mean TGF-α content for lung tumours is only 2 times higher than the normal parenchyma, the mean T/N ratio for paired specimens is $4.1 \pm 1.02$ (versus at least $7.12 \pm 1.84$ for colon), and only 33% of lung carcinomas demonstrate a T/N ratio of greater than 4. However, similar to colon cancers, the TGF-α levels cannot be correlated to the histological types of tumour, the stage of the disease, nor the degree of desmoplastic reaction in the tumours. Bergh (1988) has also reported that expression of TGF-α alone in non-small cell lung carcinoma cell lines could not be correlated with the extent of fibrosis in tumours formed by these cells in nude mice.

TGF-α has been considered as one of the prototypes of an autocrine growth factor which may play important roles in carcinogenesis and tumour growth (Sporn & Todaro, 1980; Sporn & Roberts, 1985; Goustin *et al.*, 1986; Derynck, 1988). TGF-α may also play an important role in tumour angiogenesis (Schreiber *et al.*, 1986), and in the pathogenesis of paraneoplastic hypercalcaemia (Ibbotson *et al.*, 1986; Tashjian *et al.*, 1985). Nonetheless, the importance of TGF-α in tumour cell biology *in vivo* remains speculative, and our own analyses on colon and lung carcinomas have not provided further insights. Rothbauer *et al.* reported that in 2 of 3 patients with familial polyposis, the TGF-α levels in either the preneoplastic adenomatous polyps, or carcinoma, were actually lower than those in the 'normal' mucosa, suggesting that TGF-α expression is not related to the mechanism of multistage carcinogenesis. The introduction and expression of hTGF-α gene into mouse keratinocytes from normal skin or papillomas did not result in the formation of carcinoma, suggesting that this growth factor does not influence tumour progression directly (Finzi *et al.*, 1988). However, correlation of TGF-α synthesis or secretion with tumorigenicity and the pathological stage also cannot be demonstrated in human breast cancer cell lines/tissues (Dickson *et al.*, 1986; Perroteau *et al.*, 1986; Zajchowski *et al.*, 1988; Ciardiello *et al.*, 1989). The marked heterogeneity of TGF-α expression, even among cancers of the same organ or histological type, suggests that it is unlikely to play a consistent and direct role in the pathogenesis of all cancers. However, TGF-α may still play an important indirect but synergistic role in the tumour biology of some individual human cancers, especially through its autocrine effects on cell proliferation and tumour growth rate, on pericellular matrix proteolysis involving the plasminogen activator systems (Laiho & Keski-Oja, 1989), and through its paracrine effect on angiogenesis (Schreiber *et al.*, 1986). Further knowledge on the level and pattern of expression of TGF-α in primary human cancers may also contribute understanding to its future use as a tumour marker (Yeh *et al.*, 1987; Ciardiello *et al.*, 1989), prognostic indicator (Arteaga *et al.*, 1988) and therapeutic target (Greig *et al.*, 1988).

We gratefully thank Dr David Lee (University of North Carolina at Chapel Hill) and Dr S.-Y. Mah (Royal Victoria Hospital, Montreal) for their generosity in providing respectively the rat TGF-α and human β-actin cDNA probes. This work was supported by the Cancer Research Society, Inc. of Montreal, and Dr Liu is the recipient of a fellowship from this society. Anna Woo was supported by a summer research bursury from the faculty of medicine of McGill University. Dr Tsao is a scholar of the Medical Research Council of Canada.

### References

ANZANO, M.A., ROBERTS, A.B., SMITH, J.M. & 2 others (1983). Sarcoma growth factor from conditioned medium of virally transformed cells is composed of both type α and type β transforming growth factors. *Proc. Natl Acad. Sci. USA*, **80**, 6264.

ANZANO, M.A., RIEMAN, D., PRICHETT, W. & 2 others (1989). Growth factor production by human colon carcinoma cell lines. *Cancer Res.*, **49**, 2898.

ARTEAGA, C.L., HANAUSKE, A.R., CLARK, G.M. & 5 others (1988). Immunoreactive α transforming growth factor activity in effusions from cancer patients as a marker of tumor burden and patient prognosis. *Cancer Res.*, **48**, 5023.

BERGH, J. (1988). The expression of the platelet-derived and transforming growth factor genes in human nonsmall lung cancer cell lines is related to tumor stroma formation in nude mice tumors. *Am. J. Pathol.*, **133**, 434.

BENNETT, C., PATERSON, I.M., CORBISHLEY, C.M. & 1 other (1989). Expression of growth factor and epidermal growth factor receptor encoded transcripts in human gastric tissues. *Cancer Res.*, **49**, 2104.

CARPENTER, G., STOSCHECK, C.M., PRESTON, Y.A. & 1 other (1983). Antibodies to the epidermal growth factor receptor block the biological activities of sarcoma growth factors. *Proc. Natl Acad. Sci. USA*, **80**, 5627.

CARTLIDGE, S.A. & ELDER, J.B. (1989). Transforming growth factor α and epidermal growth factor levels in normal human gastrointestinal mucosa. *Br. J. Cancer*, **60**, 657.

CHURCH, G.M. & GILBERT, W. (1984). Genomic sequencing. *Proc. Natl Acad. Sci. USA*, **81**, 1991.

CIARDIELLO, F., KIM, N., LISCIA, D.S. & 9 others (1989). mRNA expression of transforming growth factor alpha in human breast carcinomas and its activity in effusions of breast cancer patients. *J. Natl Cancer Inst.*, **81**, 1165.

COFFEY, R.J., GOUSTIN, A.S., SODERQUIST, A.M. & 4 others (1987*a*). Transforming growth factor α and β expression in human colon cancer lines: implication for an autocrine model. *Cancer Res.*, **47**, 4590.

COFFEY, R.J., DERYNCK, R., WILCOX, J.N. & 4 others (1987*b*). Production and auto-induction of transforming growth factor-α in human keratinocytes. *Nature*, **328**, 817.

DELARCO, J.E., TODARO, G.J. (1978). Growth factors from murine sarcoma virus-transformed cells. *Proc. Natl Acad. Sci. USA*, **75**, 4001.

DERYNCK, R., ROBERTS, A.B., WINKLER, M.E. & 2 others (1984). Human transforming growth factor-α: precursor structure and expression in E. coli. *Cell*, **38**, 287.

DERYNCK, R., GOEDDEL, D.V., ULLRICH, A. & 4 others (1987). Synthesis of messenger RNAs for transforming growth factor α and β and the epidermal growth factor receptor by human tumors. *Cancer Res.*, **47**, 707.

DERYNCK, R. (1988). Transforming growth factor α. *Cell*, **54**, 593.

DICKSON, R.B., BATES, S.E., MCMANAWAY, M.E. & 1 other (1986). Characterization of estrogen responsive transforming activity in human breast cancer cell lines. *Cancer Res.*, **46**, 1707.

FINZI, E., KILKENNY, A., STRICKLAND, J.E. & 5 others (1988). TGF-α stimulates growth of skin papilloma by autocrine and paracrine mechanisms but does not cause neoplastic progression. *Molec. Carcinogenesis*, **1**, 7.

GOMELLA, L.G., SARGENT, E.R., WADE, T.P. & 3 others (1989). Expression of transforming growth factor α in normal human adult kidney and enhanced expression of transforming growth factors α and β1 in renal cell carcinoma. *Cancer Res.*, **49**, 6972.

GOUSTIN, A.E., LEOF, E.B., SHIPLEY, G.D. & 1 other (1986). Growth factors and cancer. *Cancer Res.*, **46**, 1015.

GREIG, R., DUNNINGTON, D., MURTHY, U. & 1 other (1988). Growth factors as novel therapeutic targets in neoplastic disease. *Cancer Surveys*, **7**, 653.

HANAUSKE, A.R., BUCHOK, J., SCHEITHAUER, W. & 1 other (1987). Human colon cancer cell lines secreted α TGF-like activity. *Br. J. Cancer*, **55**, 57.

IBBOTSON, K.J., HARROD, J., GOWEN, M. & 5 others (1986). Human recombinant transforming growth factor α stimulates bone resorption and inhibits formation in vitro. *Proc. Natl Acad. Sci. USA*, **83**, 2228.

LAIHO, M. & KESKI-OJA, J. (1989). Growth factors in the regulation of pericellular proteolysis: a review. *Cancer Res.*, **49**, 2533.

LEE, D.C., ROSE, T.M., WEBB, N.R. & 1 other (1985). Cloning and sequence analysis of a cDNA for rat transforming growth factor-α. *Nature*, **313**, 489.

MADTES, D.K., RAINES, E.W., SAKARIASSEN, K.S. & 4 others (1988). Induction of transforming growth factor-α in activated human alveolar macrophages. *Cell*, **53**, 285.

MARQUARDT, H., HUNKAPILLER, M.W., HOOD, L.E. & 4 others (1983). Transforming growth factors produced by retrovirus-transformed rodent fibroblasts and human melanoma cells: amino acid sequence homology with epidermal growth factor. *Proc. Natl Acad. Sci. USA*, **80**, 4684.

MARQUARDT, H., HUNKAPILLER, M.W., HOOD, L.E. & 1 other (1984). Rat transforming growth factor type 1: structure and relation to epidermal growth factor. *Science*, **223**, 1079.

MCCLARE, J.T. & DIETRICH II, F.H. (1988). *Statistics*. Dellen Publishing Co.: San Francisco.

NICKELL, K.A., HALPER, J. & MOSES, H.L. (1983). Transforming growth factors in solid human malignant neoplasms. *Cancer Res.*, **43**, 1966.

PONTE, P., GUNNING, P., BLAU, H. & KEDES, L. (1983). Human actin genes are single copy for α-skeletal and α-cardiac actin but multicopy for β- and -cytoskeletal genes: 3′ untranslated regions are isotype specific but are conserved in evolution. *Mol. Cell. Biol.*, **3**, 1783.

PORROTEAU, I., SALOMON, D., DEBORTOLI, M. & 5 others (1986). Immunological detection and quantitation of α transforming growth factors in human breast carcinoma cells. *Breast Cancer Res. Treat.*, **7**, 201.

RAPPOLEE, D.A., MARK, D., BANDA, M.J. & 1 other (1988). Wound macrophages express TGF-α and other growth factors in vivo: analysis by mRNA phenotyping. *Science*, **241**, 708.

ROBERTS, A.E., LAMB, L.C., NEWTON, D.L. & 2 others (1980). Transforming growth factors: isolation of polypeptides from virally and chemically transformed cells by acid/ethanol extraction. *Proc. Natl Acad. Sci. USA*, **77**, 3494.

ROTHBAUER, E., MANN, K., WIEBECKE, B. & 5 others (1989). Epidermal growth factor receptors and epidermal growth factor-like activity in colorectal mucosa, adenomas and carcinomas. *Klin. Wochenschr.*, **67**, 518.

SCHREIBER, A.B., WINKLER, M.E. & DERYNCK, R. (1986). Transforming growth factor-α is a more potent angiogenic mediator than epidermal growth factor. *Science*, **232**, 1250.

SPORN, M.B. & TODARO, G.J. (1980). Autocrine secretion and malignant transformation of cells. *New Engl. J. Med.*, **303**, 878.

SPORN, M.B. & ROBERTS, A.B. (1985). Introduction: autocrine, paracrine and endocrine mechanisms of growth control. *Cancer Surveys*, **4**, 627.

TASHJIAN, A.H.J., VOELKEL, E.F., LAZZARO, M. & 5 others (1985). α and β human transforming growth factors stimulate prostaglandin production and bone resorption in cultured mouse calvaria. *Proc. Natl Acad. Sci. USA*, **82**, 4535.

WATKINS, L.F., BRATTAIN, M.G. & LEVINE, A.E. (1988). Modulation of a high molecular weight form of transforming growth factor-α in human colon carcinoma cell lines. *Cancer Lett.*, **40**, 59.

ZAJCHOWSKI, D., BAND, V., PAUZIE, N. & 3 others (1988). Expression of growth factors and oncogenes in normal and tumor-derived human mammary epithelial cells. *Cancer Res.*, **48**, 7041.

YEH, Y.-C., TSAI, J.-F., CHUANG, L.-Y. & 4 others (1987). Elevation of transforming growth factor α and its relationship to the epidermal growth factor and α-fetoprotein levels in patients with hepatocellular carcinoma. *Cancer Res.*, **47**, 896.